# Risk factors for fecal carriage of drug-resistant *Escherichia coli*: a systematic review and meta-analysis

**DOI:** 10.1186/s13756-020-0691-3

**Published:** 2020-02-11

**Authors:** Yuan Hu, Yusuke Matsui, Lee W. Riley

**Affiliations:** 1 School of Public Health, Division of Epidemiology, University of California Berkeley, 530E Li Ka Shing, Berkeley, 94720 CA USA; 2 School of Public Health, Division of Infectious Disease and Vaccinology, University of California Berkeley, 530E Li Ka Shing, Berkeley, 94720 CA USA

**Keywords:** Drug resistance, Commensal *Escherichia coli*, Risk factors, Systematic review, Meta-analysis

## Abstract

**Background:**

Antimicrobial resistance is a serious public health problem. Fecal carriage of drug-resistant bacteria has been suggested as an important source of antimicrobial resistant genes (ARGs). We aimed to identify risk factors associated with fecal carriage of drug-resistant commensal *Escherichia coli* among healthy adult population.

**Methods:**

We conducted a systematic review and meta-analysis following the PRISMA guideline. We identified observational studies published from 2014 to 2019 through PubMed, Embase, and Web of Science. Studies were eligible if they investigated and reported risk factors and accompanying measure of associations for fecal carriage of drug-resistant *E. coli* for healthy population aged 18-65. Data on risk factors assessed in three or more studies were extracted.

**Results:**

Fifteen of 395 studies involving 11480 healthy individuals were included. The pooled prevalence of drug-resistant Enterobacteriaceae was 14% (95% confidence interval [CI] 8-23%). Antimicrobial use within the 12 months prior to stool culture (odds ratio [OR] 1.84 [95%CI 1.35-2.51]), diarrhea symptoms (OR 1.56 [95%CI 1.09-2.25]), travel to India (OR 4.15 [95%CI 2.54-6.78]), and vegetarian diet (OR 1.60 [95%CI 1.00(1.0043)-2.56(2.5587)]) were associated with increased risk of fecal carriage of drug-resistant *E. coli*. Among travellers, antimicrobial use (OR 2.81 [95%CI 1.47-5.36]), diarrhea symptoms (OR 1.65 [95%CI 1.02-2.68]), travel to India (OR 3.80 [95%CI 2.23-6.47]), and vegetarian diet (OR 1.92 [95%CI 1.13-3.26]) were associated with increased risk. Among general adult population, antimicrobial use (OR 1.51 [95%CI 1.17-1.94]), diarrhea symptoms (OR 1.53 [95%CI 1.27-1.84]), and travel to Southeast Asia (OR 1.67 [95%CI 1.02-2.73]) were associated with the increased risk of drug-resistant *E. coli* carriage.

**Conclusions:**

The findings indicate that dietary habit as well as past antimicrobial use and travel to high-risk country are associated with the risk of fecal carriage of drug-resistant commensal *E. coli*.

## Background

Antimicrobial resistance is one of the most pressing public health challenges of our time. In particular, the rising incidence of infections caused by drug-resistant Gram-negative bacteria is a serious problem due to the potential for rapid spread of resistance via mobile elements and limited treatment options [1–3].Among Gram-negative bacteria developing drug resistance, *Escherichia coli* (*E. coli*) is the most frequent cause of extraintestinal infections such as urinary tract infection and bloodstream infection [2]. Drug-resistant intestinal pathogenic *E. coli*, such as Shiga toxin-producing *E. coli* (STEC), are also increasingly recognized [4, 5]. *E. coli* can be transmitted through contaminated water or food, or through contact with people and other animals [6]. The prevalence and incidence of infections caused by drug-resistant pathogenic *E. coli* have been rapidly increasing worldwide [2, 7, 8].

Major sources of drug-resistant bacteria include the environment such as contaminated water [9], food including meat [10, 11] and vegetables [12, 13], and healthcare settings [14]. Additionally, intestinal commensal drugresistant bacteria have been reported as an important reservoir of antimicrobial drug resistance genes (ARGs) [15, 16]. Surveillance on human fecal carriage of drug-resistant bacteria has revealed that there is an increasing trend in intestinal ARG carriage worldwide [7, 17].

Numbers of studies have independently reported potential risk factors for the intestinal carriage of drug-resistant bacteria. Most of these studies have found previous antibiotic use to be associated with drug-resistant bacteria carriage in both primary care patients and healthy populations [18, 19]. Also, traveling to developing countries has been identified as a risk factor for acquiring drug-resistant bacteria [20]. Risk factors related to healthcare-associated infections (HAI) have been reported as well, including admission to the intensive care unit (ICU), use of catheter, and dialysis [21–23].

*E. coli* is also a member of the commensal flora of human and other warm-blooded animal intestinal tracts. As such, they can acquire ARGs by horizontal gene transfer [24] from drug-resistant *E. coli* strains and other Gram-negative bacteria that enter the intestinal tract via exposures to contaminated food, water, and other external sources. Thus, risk factors for fecal carriage of drug-resistant commensal *E. coli* and ARGs could include exposures to environmental sources of drug-resistant bacteria, in addition to traditional risks such as prior use of antibiotics.

The impact or magnitude of exposures to food on the commensal *E. coli* carriage of ARGs is not known. Identifying risk factors for fecal carriage of drug-resistant commensal *E. coli* associated with food could potentially improve public health intervention to prevent the spread of drug-resistant *E. coli* and ARGs. While a recent review studied risk factors for fecal carriage of Gram-negative bacteria expressing extended-spectrum beta-lactamase (ESBL) reported by papers from OECD countries from 1978 to 2015 [19], there has not been a comprehensive analysis of more recent literature reporting other resistance mechanisms of human commensal *E. coli*.

The purpose of this review was to investigate risk factors associated with intestinal carriage of drug-resistant commensal *E. coli* in the recent five years. We also aimed to identify risk factors related to food. We focused on the recent five years because of the increasing prevalence of multiple mechanisms of resistance among Gram-negative bacteria causing extraintestinal and intestinal infections during this period, including mechanisms such as ESBL [25, 26], carbapenemase [27], and metallo-beta-lactamase production [26], and plasmid-mediated colistin resistance [28].

## Methods

### Data sources and search strategy

The protocol of this meta-analysis was not preregistered. We performed a systematic review and meta-analysis following the PRISMA [Preferred Reporting Items for Systematic Reviews and Meta Analyses] guidelines [29] (Additional file 2: Table S1). We conducted a literature search with the databases PubMed, Embase, and Web of Science. We limited the search to articles published between 2014 and 2019. Only articles published in English were included. The search focused on risk factors for intestinal carriages of drug-resistant commensal *E. coli*, which was conducted on August 9th, 2019. For the purpose of this review, the definition of antimicrobial drug resistance was based on the drug-susceptibility test results (disk diffusion test, minimum inhibitory concentration (MIC) test, VITEK) reported by the clinical microbiology or research laboratories described in the reviewed studies, which followed the guidelines of organizations such as the Clinical and Laboratory Standards Institute (CLSI). We included reports of *E. coli* resistance to beta-lactams, aminoglycosides, fluoroquinolones, and tetracyclines. We included search terms: (feces [Title/Abstract] OR stool [Title/Abstract] OR fecal [Title/Abstract] OR faecal [Title/Abstract] OR “rectal swab” [Title/Abstract]) AND (“escherichia coli” [Title/Abstract] OR escherichia [Title/Abstract] OR “e.coli” [Title/Abstract])) AND (“drug resistant” [Title/Abstract] OR “drug susceptible” [Title/Abstract] OR “drug susceptibility” [Title/Abstract] OR “antimicrobial resistance” [Title/Abstract] OR “antimicrobial resistant” [Title/Abstract] OR resistant [Title/Abstract] OR resistance [Title/Abstract] OR drug [Title/Abstract] OR multidrug [Title/Abstract]) AND (questionnaire [Title/Abstract] OR surveillance [Title/Abstract] OR survey [Title/Abstract]).

After the databases were reviewed, the results were exported and then compiled with the reference management software Covidence [30]. Duplicates were removed by automated process of Covidence, followed by a manual search to identify and remove additional duplicates.

### Study selection

All abstracts were screened first by author YH and then by author YM to minimize omission of eligible studies. Screening criteria were as follows: (1) examined bacteria must include *E. coli* or Enterobacteriaceae; (2) examined bacteria must be isolated from human feces, stool, or rectal swab; (3) must report risk factors. Studies reporting risk factors for drug-resistant Enterobacteriaceae were considered eligible because *E. coli* is the most common Enterobacteriaceae. Studies that remained of interest were then screened based on their full text by two independent reviewers, YH and YM. Disagreements were resolved by consensus. Inclusion criteria were: (1) reported risk factor(s); (2) reported measure of associations and accompanying 95% confidence intervals (95% CI) or its equivalent; (3) study population aged 18-65; (4) healthy study population; (5) survey conducted after 2010.

For the meta-analysis, we excluded studies that (1) did not report risk factors commonly assessed in 3 or more studies or (2) did not offer sufficient data to create a contingency table.

### Data extraction

Data were first extracted by YH and checked by YM. The assessment measures extracted from the included studies were as follows: (1) publication data: lead author names, year of publication; (2) demographic and epidemiological data: location of study, study population, study design, sample size, outcome, prevalence of drug-resistant bacteria, outcome measurement methods, statistical analysis methods; (3) risk-factor associated data: risk factor(s) investigated, measure of associations (odds ratios, risk ratios or prevalence ratios) and accompanying 95% CI.

When enumerating risk factors from each eligible study, we did not limit the analysis to statistically significant factors to avoid publication bias and to identify as many factors studied to date as possible.

### Meta-analysis

For studies which provided enough data to allow for the creation of contingency tables, unless the authors reported an adjusted OR and corresponding 95% CI, we manually calculated the OR and 95% CI. If there were insufficient data to create a contingency table, we excluded the study to calculate pooled estimates.

We performed random-effects meta-analysis under a Mantel-Haenszel model with Hartung-Knapp adjustment to estimate the pooled effect of each commonly reported risk factors for intestinal carriage of drug-resistant *E. coli*. Mantel-Haenszel random-effects model estimates the amount of between-study variation by comparing each study’s result with a fixed-effect meta-analysis result but avoids approximating Normal distributions [31, 32]. Hartung-Knapp adjustment provides a more conservative and robust pooled OR estimates and 95% CI, allowing for any heterogeneity between studies even when the study number is small and study size is unequal [33]. Forest plots were created to visualize the reported OR and 95% CI from each studies and pooled ORs for each commonly assessed risk factors. We assessed statistical heterogeneity between studies by the *Chi*^2^ test and variation due to heterogeneity across the studies by the *I*^2^ statistic. *P*<0.10 was considered indicative of statistically significant heterogeneity in the *Chi*^2^ test, and *I*^2^ values of 25, 50 and 75% were defined as low, moderate, and high estimates, respectively. We evaluated the potential for publication bias with funnel plots and Egger’s tests for meta-analyses with at least 10 studies [34], which test for asymmetry of the funnel plot and effects of small studies. Analyses were conducted with R version 3.5.1 [35], with package ’meta’ version 4.9-6 [36].

## Results

### Study selection

Our search identified 395 unique studies that we assessed for eligibility with title and abstract screening. Of these, 58 studies were forwarded to full-text article screening. Of the 58 full-text articles, we identified 15 relevant articles that reported risk factors associated with drug-resistant Enterobacteriaceae (10) or *E. coli* (5) carriage [37–51].

**Fig. 1:**
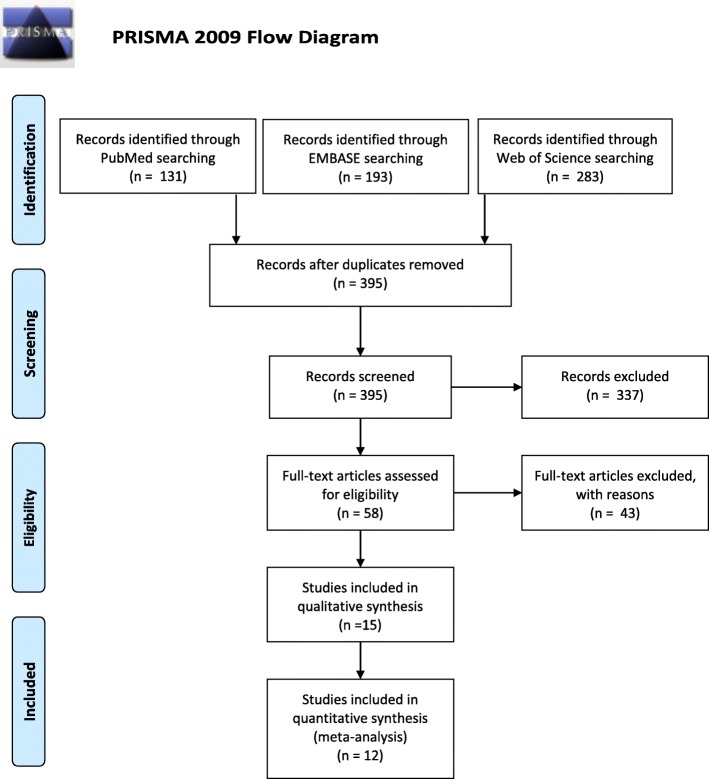
PRISMA Flow Diagram. Flow diagram of the systematic review process used to identify eligible studies

Twelve of 15 studies included in the systematic review were eligible for inclusion in the meta-analysis, which reported sufficient data to create contingency tables to compare risk factors that were studied in at least three of the studies [37, 38, 40, 42–48, 50, 51]. Caudell et al. (2018) did not report risk factors commonly assessed in 3 or more studies and Dohmen et al. (2017) and Sanneh et al. (2018) did not offer sufficient data to create a contingency table [39, 41, 49]. See Fig. 1, Table 1, and Additional file 3: Table S2 for further details of search and reasons for exclusion.

**Table 1:** Characteristics of studies included in review, 2014-2019

Author, year	Country	Study population	Study design	Study period	Sample size	Pathogen type
Arcilla 2017	Netherlands	Travellers	Prospective cohort study	2012 Nov - 2013 Nov	1847	ESBL-PE, CPE
Angelin 2015	Sweden	Travellers	Prospective study	2010 Apr - 2014 Jan	99	*E. coli*
Caudell 2018*	Tanzania	General adult	Prospective study	2012 Mar - 2015 Jul	226*	*E. coli*
Dohmen 2017	Netherlands	Employees in a pig slaughterhouse	Prospective study	2015 Jun	334	*E. coli*
Dohmen 2017*	Netherlands	Pig farmers, family members and employees	Longitudinal study	2011 Mar - 2011 Oct	146	ESBL-PE
Lubbert 2015	Germany	Travellers	Prospective cohort study	2013 May - 2014 Apr	191	ESBL-PE
McNulty 2018	England	General adult	Retrospective cohort study	2013 - 2014	2430	ESBL-PE
Miranda 2016	Germany	Travellers	Retrospective study	2013 Feb - 2014 Apr	211	ESBL-PE
Mo 2019	Singapore	General adult	Cross sectional study	2016 Jun - 2017 Apr	305	ESBL-PE
Reuland 2016	Netherlands	General adult	Case control study	2011 Jun - 2011 Nov	1695	ESBL-PE
Reuland 2015	Netherlands	General adult	Case control study	2011 Aug - 2011 Dec	550	pAmpC producing *E. coli*
Ruh 2019	Northern Cyprus	General adult	Retrospective cohort study	2017 Sep - 2017 Dec	500	Enterobacteriaceae
Sanneh 2018*	Gambia	Food handlers	Cross sectional study	2015 Jul - 2015 Sep	565	Enterobacteriaceae
Vading 2016	Sweden	Travellers	Prospective cohort study	2013 Apr - 2015 May	175	ESBL *E. coli*
Wielders 2017	Netherlands	General adult	Cross sectional study	2012 Nov	2432	ESBL-PE

Note: ** not included in meta-analysis. *indicates sample size was households (all others are individuals). ESBL-PE = Extended-spectrum beta-lactamase producing Enterobacteriaceae; CPE = Carbapenemase-producing Enterobacteriaceae

### Study characteristics

The 15 studies represented 8 countries: England, Gambia, Germany, Netherlands, Northern Cyprus, Singapore, Sweden, and Tanzania (Table 1). None of the studies reported randomization in participant selection. Eight studies sampled volunteers from healthy general population that were registered to a hospital system. Five were cohort studies of healthy travellers that compared the prevalence of drug-resistant Enterobacteriaceae or *E. coli* before and after the travel. Two studies surveyed pig farmers.

Five studies reported prevalence of drug-resistant *E. coli*, while 10 studies investigated Enterobacteriaceae. The frequency of *E. coli* among Enterobacteriaceae ranged from 79-97% for 9 studies, while one study reported 29%. All studies collected information on demographic factors, behaviors, and past illness from participants. Some studies excluded insufficient response from the surveys.

**Fig. 2:**
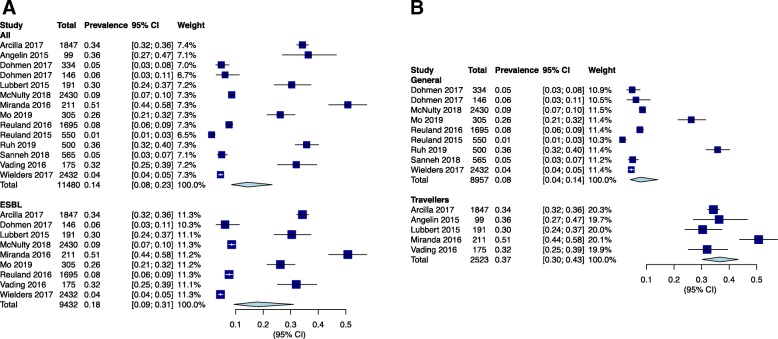
Forest plots for individuals and combined prevalence estimates of fecal carriage of drug-resistant bacteria. **a** Prevalence of drug-resistant Enterobacteriaceae and prevalence of ESBL-producing Enterobacteriaceae; **b** Prevalence of drug-resistant Enterobacteriaceae among travellers and general populations

The prevalence of fecal drug-resistant Enterobacteriaceae reported in the studies ranged from 1% to 51%. The pooled prevalence was 14% (95% CI 8-23%) (Fig. 2a). Nine studies reported ESBL producing Enterobacteriaceae. The pooled prevalence of ESBL-producing Enterobacteriaceae was 18% (95%CI 9-31%) (Fig. 2a). The prevalence among general population was 8% (95%CI 4-14%) (Fig. 2b) and among travellers was 37% (95%CI 30-43%) (Fig. 2b). All studies followed established drug susceptibility testing methods, disc diffusion tests, VITEK 2, or minimum inhibitory concentration (MIC) measurement. Common statistical methods for risk factor analysis included univariate and multivariate logistic regression, chi-squared test, and Fisher’s exact *t* test.

### Commonly assessed risk factors

**Fig. 3:**
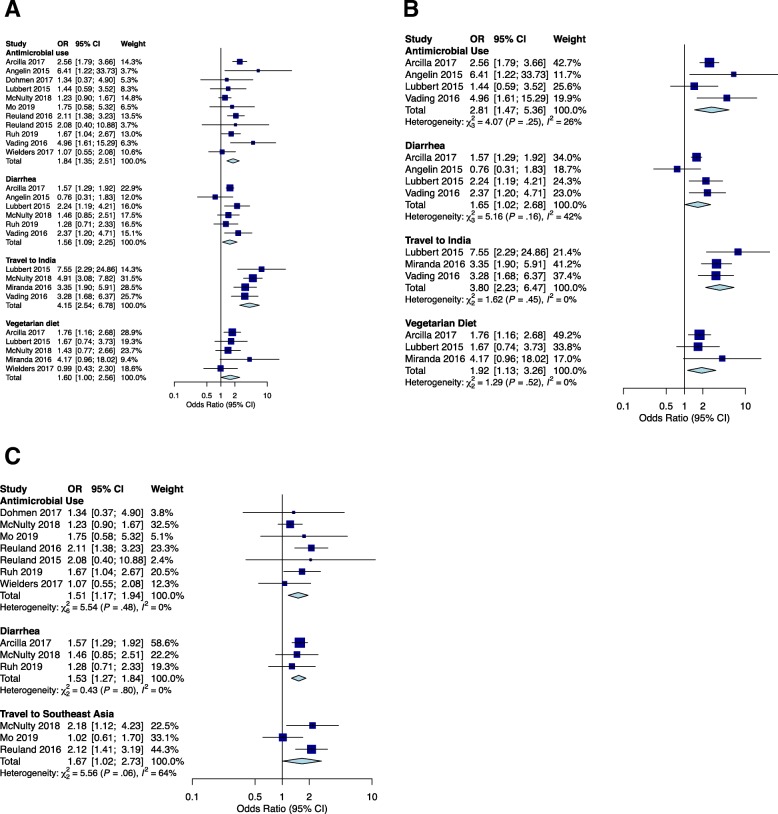
Forest plots for significant risk factors. **a** Individuals and combined OR of fecal carriage of drug-resistant *E. coli* among entire population; **b** Individuals and combined OR of fecal carriage of drug-resistant *E. coli* among travellers; **c** Individuals and combined OR of fecal carriage of drug-resistant *E. coli* among general population. OR, odds ratio

Commonly assessed risk factors identified in this review are shown in Table 2. We identified fourteen risk factors assessed in three or more studies. We assessed the pooled ORs in the meta-analysis (Table 2, Fig. 3a, Additional file 1: Figure S1a).

**Table 2:** Commonly assessed risk factors for intestinal carriage of drug-resistant *E. coli*, 2014-2019. OR = Odds Ratio; CI = Confidence interval. Note: *indicates results from systematic review

Risk factor	Number of studies investigated*	Number of studies finding significant association*	Number of samples assessed	Number of samples with drug resistant bacteria	Pooled OR (95%CI)	*χ*^2^(P-value)	*l*^2^
General factors							
Gender	11	1	9836	1428	1.16 (0.98-1.36)	8.77 (0.46)	0
Diet restriction (vegetarian)	5	2	6802	989	1.60 (1.00-2.56)	3.22 (0.52)	0
Pet	4	1	5159	407	1.15 (0.33-4.06)	5.23 (0.16)	43
Education level	4	0	5067	925	0.93 (0.74-1.17)	0.98 (0.81)	0
Smoking	4	1	4497	712	0.77 (0.18-3.25)	6.37 (0.04)	69
Clinical factors							
Antimicrobial use	13	6	10079	1407	1.84 (1.35-2.51)	18.28 (0.05)	45
Previous hospital admission	7	2	6108	465	1.63 (0.84-3.18)	7.83 (0.17)	36
Diarrhea	7	4	5144	1079	1.56 (1.09-2.25)	5.76 (0.33)	13
Proton-pump inhibitor use	3	2	4111	359	1.31 (0.11-15.5)	5.81 (0.05)	66
Chronic disease	3	2	2323	766	0.91 (0.13-6.53)	8.68 (0.01)	77
Travel related factors							
International travel	6	2	6460	520	1.13 (0.67-1.91)	10.73 (0.06)	53
Travel to Southeast Asia	8	4	6632	1289	1.78 (0.64-4.98)	50.28 (<0.01)	86
Travel to Africa	5	2	6692	1105	1.29 (0.52-3.21)	81.34 (<0.01)	94
Travel to India	4	4	2953	423	4.15 (2.54-6.78)	2.50 (0.48)	0

OR = Odds Ratio; CI = Confidence interval. Note: *indicates results from systematic review

Traveling to India was the only risk factor that all studies reported to be significantly associated with fecal carriage of drug-resistant *E. coli*. For the remaining risk factors, ORs and accompanying 95% CI were found to vary among studies. There were three risk factors that showed significant pooled ORs. These included antimicrobial use within the previous 12 months (OR 1.84 [95% CI 1.35-2.51]), diarrhea symptoms (OR 1.56 [95% CI 1.09-2.25]), and vegetarian diet (OR 1.60 [95% CI 1.00(1.0043)-2.56(2.5587)]). Six (46%) of 13 studies found antimicrobial use in the previous 12 months, 4 (57%) of 7 studies found diarrhea symptom, and 2 (40%) of 5 studies found vegetarian diet to be significantly associated with the carriage of drug-resistant bacteria.

Smoking, living with pet(s), gender, education level, previous hospital admission, proton-pump inhibitor (PPI) use, chronic disease, international travel, travel to Southeast Asia and exposure to livestock were commonly assessed but no significant pooled OR was found in these studies. Of these commonly assessed risk factors, three factors (PPI use, chronic disease, travel to Southeast Asia) were reported as significant risks among half or more studies included in this review. Two (67%) of 3 studies found PPI use, 2 (67%) of 3 studies found chronic disease, and 4 (50%) of 8 studies found travel to Southeast Asia to be significantly associated with the carriage of drug-resistant bacteria.

### Risk factors based on travelling status

The prevalence of drug-resistant *E. coli* carriage suggested two distinct populations. We divided the population into travellers and other general population adults and replicated the analysis (Table 3, Fig. 3b, c, and Additional file 1: Figure S1b, c). Antimicrobial use within the previous 12 months, diarrhea symptoms, gender, travelling to India, travelling to Africa, and travelling to Southeast Asia were assessed for travellers. We also assessed antimicrobial use within the previous 12 months, diarrhea symptoms, gender, travelling abroad, travelling to Southeast Asia, education status, pet, and previous hospitalization among general population adults. The results showed that antimicrobial use within the previous 12 months (OR 2.81 [95% CI 1.47-5.36]), diarrhea symptoms (OR 1.65 [95% CI 1.02-2.68]), vegetarian diet (OR 1.92 [95% CI 1.13-3.26]), and travelling to India (OR 3.80 [95% CI 2.23-6.47]) remained significant risk factors among travellers. Among general population adults, antimicrobial use within the previous 12 months (OR 1.51 [95% CI 1.17-1.94]), diarrhea symptoms (OR 1.53 [95% CI 1.27-1.84]), and travelling to Southeast Asia (OR 1.67 [95% CI 1.02-2.73]) were significant risk factors.

**Table 3:** Commonly assessed risk factors for intestinal carriage of drug-resistant *E. coli*, 2014-2019, stratified by travellers and general adults

	Travellers				General adults			
Risk Factor	Number of studies investigated*	Pooled OR (95%CI)	*χ*^2^(P-value)	*l*^2^(*%*)	Number of studies investigated*	Pooled OR (95%CI)	*χ*^2^(P-value)	*l*^2^(%)
General factors								
Gender	4	1.14 (0.85-1.51)	2.17(0.54)	0	6	1.16 (0.90-1.50)	6.15 (0.29)	19
Diet restriction (vegetarian)	3	1.92 (1.13-3.26)	1.29 (0.52)	0	1	-	-	-
Pet	1	-	-	-	3	0.93 (0.70-1.24)	0.94 (0.63)	0
Education level	1	-	-	-	3	0.92 (0.63-1.35)	0.98 (0.81)	0
Clinical factors								
Antimicrobial use	4	2.81 (1.47-5.36)	4.07 (0.25)	26	7	1.51 (1.17-1.94)	5.54 (0.48)	0
Previous hospital admission	1	-	-	-	5	1.47 (0.79-2.76)	5.54 (0.24)	28
Diarrhea	4	1.65 (1.02-2.68)	5.16 (0.16)	42	3	1.53 (1.27-1.84)	0.43 (0.80)	0
Travel related factors								
International travel	0	-	-	-	6	1.13 (0.73-1.74)	10.73 (0.06)	53
Travel to Southeast Asia	5	1.93 (0.46-8.12)	41.24 (<0.01)	90	8	1.67 (1.02-2.73)	5.56 (0.06)	64
Travel to Africa	3	0.75 (0.29-1.96)	19.27 (<0.01)	90	2	-	-	-
Travel to India	3	3.80 (2.23-6.47)	1.62 (0.45)	0	1	-	-	-

OR = Odds Ratio; CI = Confidence interval. Note: *indicates results from systematic review

### Risk factors related to food

Six of 15 studies reported risk factors related to food. Five studies assessed the risk among vegetarians (Table 2). As stated above, pooled OR showed significant association with being a vegetarian (OR 1.60 [95% CI 1.00-2.56]). Two studies reported significant association, one with unadjusted OR [37], and another with adjusted OR [44].

Four studies reported potential food-associated risk factors other than being a vegetarian. One study reported exposure to raw milk as significant risk factor for acquiring multi-drug-resistant *E.coli* (OR 7.54 [95% CI 2.41-23.45]) [39]. Two studies reported the effect of eating street food during travel. One of them was reported as significant risk (OR 2.09 [95% CI 1.30-3.38] for daily consumption; OR was 1.37 [95% CI 1.08-1.73] for occasional consumption during travel) [37]. Another study did not find significant association (OR 0.92 [95% CI 0.49-1.74]) [42]. Two studies assessed the effect of raw vegetable consumption on the fecal carriage of drug-resistant *E. coli*. One of them reported that raw vegetable consumption during a trip to Southeast Asia significantly increased the risk of intestinal carriage of drug-resistant Enterobacteriaceae (OR 2.18 [95% CI 1.29-3.68]), while exposure to raw vegetable in South Asia significantly decreased the risk (OR 0.34 [95% CI 0.12-0.93]) [37]. The other study did not find any significant association (OR 0.58 [95% CI 0.33-1.07]) [43].

### Bias assessment and heterogeneity evaluation

**Fig. 4:**
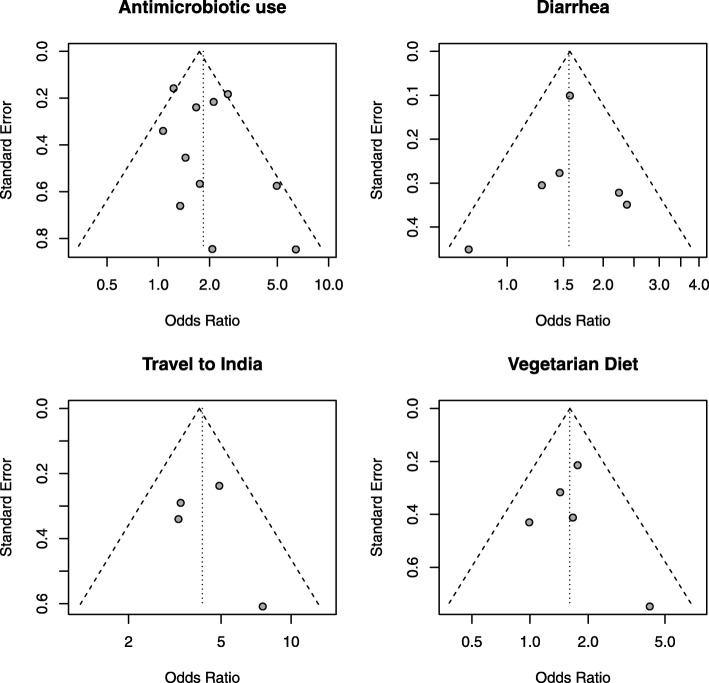
Funnel plots. Funnel plots for studies reporting antimicrobial use, diarrhea, vegetarian diet, and travel to India as risk factors

We evaluated heterogeneity among studies, and potential extent of publication bias in meta-analysis (Table 2, Table 3, Fig. 4, Fig. 3b, and c). Funnel plots of all studies reporting significant association (Fig. 4) were generated to assess the potential extent of publication bias.

For pooled estimates of all studies, risk factors related to travel showed high *chi*^2^ (11-81, *P*<0.01) and *I*^2^ value (53-94%) except for travel to India. This suggests that there was substantially high heterogeneity among studies that examined the effect of international travel, travel to Southeast Asia, and travel to Africa, respectively. Smoking, PPI use, and chronic disease status also showed moderate to high heterogeneity (*I*^2^ 66-77%). For all other risk factors, no heterogeneity was observed, suggesting that the evidence was of high quality.

For stratified estimates among travellers, travel to Africa and travel to Southeast Asia were the only risk factors that showed high heterogeneity (*chi*^2^ 19.27 and 41.24, respectively, *p*<0.01, and *I*^2^ 90%). Among general adults, travel abroad and travel to Southeast Asia showed moderate heterogeneity (*chi*^2^ 10.73 and 5.56, respectively, *p*=0.06, and *I*^2^ 53-64%). The shapes of the funnel plots were approximately symmetrical for significant risk factors, and Egger’s test showed *p*=0.42 for antimicrobial use within the previous 12 months among all populations included in this study (Fig. 4). This suggests that no publication bias existed for this factor. For all other risk factors, due to the insufficient number of studies (less than 10 studies for each), we did not evaluate the potential for publication bias with funnel plots and Egger’s tests for small study effects [34].

## Discussion

This study summarizes risk factors associated with intestinal carriage of drug-resistant Enterobacteriaceae, in particular, *E. coli* among healthy adults. Our systematic review and meta-analysis on studies published from 2014 to 2019 identified several risk factors for intestinal carriage of drug-resistant *E. coli*. We found evidence for our hypothesis that commensal *E. coli* can acquire ARGs carried by Gram-negative bacteria that enter the intestinal tract from contaminated food.

We should first note that the pooled prevalence of intestinal carriage of drug-resistant Enterobacteriaceae in our review (14% for all Enterobacteriaceae and 18% for ESBL producing Enterobacteriaceae) has slightly increased from an earlier review (14% [95% CI 9-20%] for ESBL producing Enterobacteriaceae) published in 2016 [19]. Karanika et al. conducted a systematic review and meta-analysis on papers published from 1978 to 2015 under search terms “ESBL” or “extended-spectrum beta-lactamase”, and limited the studies conducted in OECD countries. Our literature search was not limited to ESBL producing bacteria nor OECD countries. Some studies reported carbapenemase-producing Enterobacteriaceae (CPE), and extended-spectrum cephalosporin (ESC) resistant *E. coli*. High variability in the prevalence among studies could be explained by infections from external sources such as the environment, contaminated food, and contaminated water, in addition to high variability in antimicrobial usage in different regions of the world.

The high variability could also be explained by the types of populations studied. In our study, the prevalence between travellers and general adult populations were significantly different (8% [95% CI 4-14%] and 37% [95% CI 30-43%], respectively), suggesting different mechanisms for acquiring drug-resistant gut Enterobacteriaceae organisms. It is possible that travel includes distinct behavioral activities that affect exposure to potential risk factors for acquiring ARGs. This assumption led us to examine the pooled estimates of OR for each risk factor stratified by travellers vs general adult population.

In the general adult population, we found five risk factors significantly associated with intestinal carriage of drug-resistant *E. coli*, prior antimicrobial drug use within 12 months prior to stool culture, diarrhea symptoms, travel to India, travel to Southeast Asia, and vegetarian diet. Antimicrobial use, diarrhea symptoms, and travel to India were also identified in previous reports [19, 20]. When controlled by travel status, we found antimicrobial use, diarrhea, diet and travel to India significantly associated with fecal carriage of drug-resistant *E. coli* for travellers. Travel to Southeast Asia was significantly associated with ARG carriage only among the general adult population. We should note that due to the limited number of studies, some risk factors commonly assessed for entire population could not be assessed for stratified populations. To the best of our knowledge, no previous review has found vegetarian diet to be significantly associated with intestinal carriage of drug-resistant *E. coli*. Butcher et al. (2019) reported that unwashed vegetables could be a source for ESBL-producing extraintestinal pathogenic *E. coli* [52]. Multiple reports suggest association between urinary pathogenic *E. coli* and fecal *E. coli* [53, 54], and fecal carriage of drug-resistant *E. coli*. Although we should note that our pooled ORs for drug-resistant *E. coli* intestinal carriage were not controlled for potential confounding factors other than travel status, our findings suggest that certain type of dietary practice could be a risk factor for acquiring drug-resistant *E. coli* by the gut microbiota.

In addition to the five significant risk factors, we identified ten other risk factors commonly assessed in 3 or more reviewed studies. These include gender, smoking, living with pet(s), education level, proton-pump inhibitor use, previous hospital admission, chronic disease, international travel, travel to Southeast Asia, and travel to Africa. None of these factors were significantly associated with risk of intestinal carriage of drug-resistant *E. coli*. However, 50% or more of the studies reported significant associations for proton-pump inhibitor use, chronic disease, and travel to Southeast Asia. This suggests that these factors could serve as risks for drug-resistant *E. coli* colonization under certain situations. In fact, travel to Southeast Asia was a significant risk factor for general adult populations. Previous hospitalization and travel to Africa were also assessed in the review by Karanika et al. [19]. In agreement with our findings, previous hospitalization and travel to Africa were not significant risks. Stratification based on location of studies such as OECD countries to non-OECD countries and features of travel destination such as sanitation system and antibiotics usage in food production can alter the pooled ORs.

Multiple studies reported food as potential sources of *E. coli* infections [10–13, 52]. To the best of our knowledge, we found no other reviews that examined the effect of food on fecal carriage of drug-resistant *E. coli*. Being a vegetarian was significantly associated with the carriage of drug-resistant *E. coli* among overall population and travellers. Pooled estimate among general adult populations could not be obtained due to limited number of studies. Several recent studies have reported contamination of leafy green vegetables with saprophytic bacteria harboring ARGs that occur in human Gram-negative bacterial pathogens [12, 55, 56]. Four studies reported the effect of street food, raw vegetables, and raw milk consumption [37, 39, 42, 43]. However, these factors showed high variance in reported ORs among studies. This variance could be explained by differences in study region, target population, travel destination and sanitation conditions among studies. One study reported conflicting ORs for raw vegetable consumption between Southeast Asia (Brunei Darussalam, Cambodia, Indonesia, Lao People’s Democratic Republic, Malaysia, Myanmar, Philippines, Singapore, Thailand, Timor-Leste, Viet Nam) and South Asia (Afghanistan, Bangladesh, Bhutan, India, Iran (Islamic Republic of), Maldives, Nepal, Pakistan, and Sri Lanka) [37]. Geographic differences in food production methods and antimicrobial drug usage could exist. Although further studies on vegetable consumption among general population are required, this observation suggests that dietary habit can affect fecal carriage of drug-resistant *E. coli*, which supports our hypothesis that ARGs may be acquired via contaminated food in addition to healthcare-associated acquisition and person-to-person transmission.

There are limitations associated with this systematic literature review. First, 10 of 15 studies investigated Enterobacteriaceae instead of *E. coli* alone. Still, the frequency of *E. coli* found among studies that examined Enterobacteriaceae was high (79-97%) for 9 of 10 studies. One study that had low frequency (29%) of *E. coli* was not eligible for meta-analysis. Therefore, we can assume that risk factors identified in this review would apply to *E. coli*. Also, we cannot determine whether the identified risk factors have causal effects on fecal carriage of drug-resistant *E. coli*. For example, an episode of diarrhea among participants could have prompted the use of antibiotics, which could have selected for drug-resistant *E. coli* in the host intestinal microbiota. Still, identification of factors significantly associated with the carriage of drug-resistant *E. coli* will be useful for identifying individuals with high risk and early focused interventions. Another limitation of our study is that there was no study from North America included in this review. Karanika et al. (2016) reported the same limitation [19]. Since North America is a major food-exporting region in which antibiotics are heavily used in food animal husbandry and agriculture, if food is an important reservoir for drug-resistant bacteria that enter our intestines, more studies in this geographic region are needed. Also, although we did not observe publication bias for risk factors identified in this study, we found high heterogeneity among studies that reported the risk of chronic disease and travel related factors on intestinal carriage of drug-resistant bacteria. This high heterogeneity could be explained by differences in sampling methods, chronic diseases reported, travel destinations, and sanitation conditions examined in the studies. These differences could have affected the pooled OR estimates. Particularly, we should note that the chronic diseases three studies investigated were different among studies, and there was a high variation in disease incidence within the studies [37, 45, 50]. Furthermore, there were three studies reporting association for PPI use as risk factors for fecal carriage of drug-resistant *E. coli* [46, 50, 51], and McNulty et al. (2018) stated in their limitation that they did not collect data on the use of PPI [43]. Since PPI use is one of the indicators of chronic disease, larger studies related to PPI use and other chronic diseases may alter the result.

## Conclusion

In this review, we found five significant risk factors associated with intestinal carriage of drug-resistant *E. coli*, antimicrobial use, diarrhea, vegetarian diet, travel to India, and travel to Southeast Asia. Due to the high heterogeneity of the studies, other factors may indeed serve as risks under certain circumstances. Further studies, especially those that examine food and other environmental exposures will be essential for identifying public health interventions that can be devised to decrease human intestinal colonization with drug-resistant bacteria.

## Supplementary information

**Additional file 1** Forest plots of insignificant risk factors. Individuals and combined ORs of fecal carriage of drug-resistant *E. coli*. A: entire population; B: travellers; C: general adults.

**Additional file 2** PRISMA 2009 Checklist

**Additional file 3** Reference information for excluded articles and reasons for exclusion.

## Data Availability

Please contact author for data requests.

## Abbreviations

ARG: Antimicrobial resistant genes
CI: Confidence Interval
ESBL: Extended-spectrum beta-lactamase organisms
HAI: Healthcare-associated infection
ICU: Intensive care unit
OR: Odds ratio
PPI: Proton-pump inhibitor
PRISMA: Preferred reporting items for systematic reviews and meta analyses
