# Supplementary material for: Risk factors for fecal carriage of drug-resistant Escherichia coli: a systematic review and meta-analysis
Source: Antimicrob Resist Infect Control. 2020 Feb 11;9:31. doi: 10.1186/s13756-020-0691-3 (PMC7014593; doi:10.1186/s13756-020-0691-3)
Supplement: Supplementary file 1 — Additional file 1 Forest plots of insignificant risk factors. Individuals and combined ORs of fecal carriage of drug-resistant E. coli. A: entire population; B: travellers; C: general adults. [file 13756_2020_691_MOESM1_ESM.pdf]

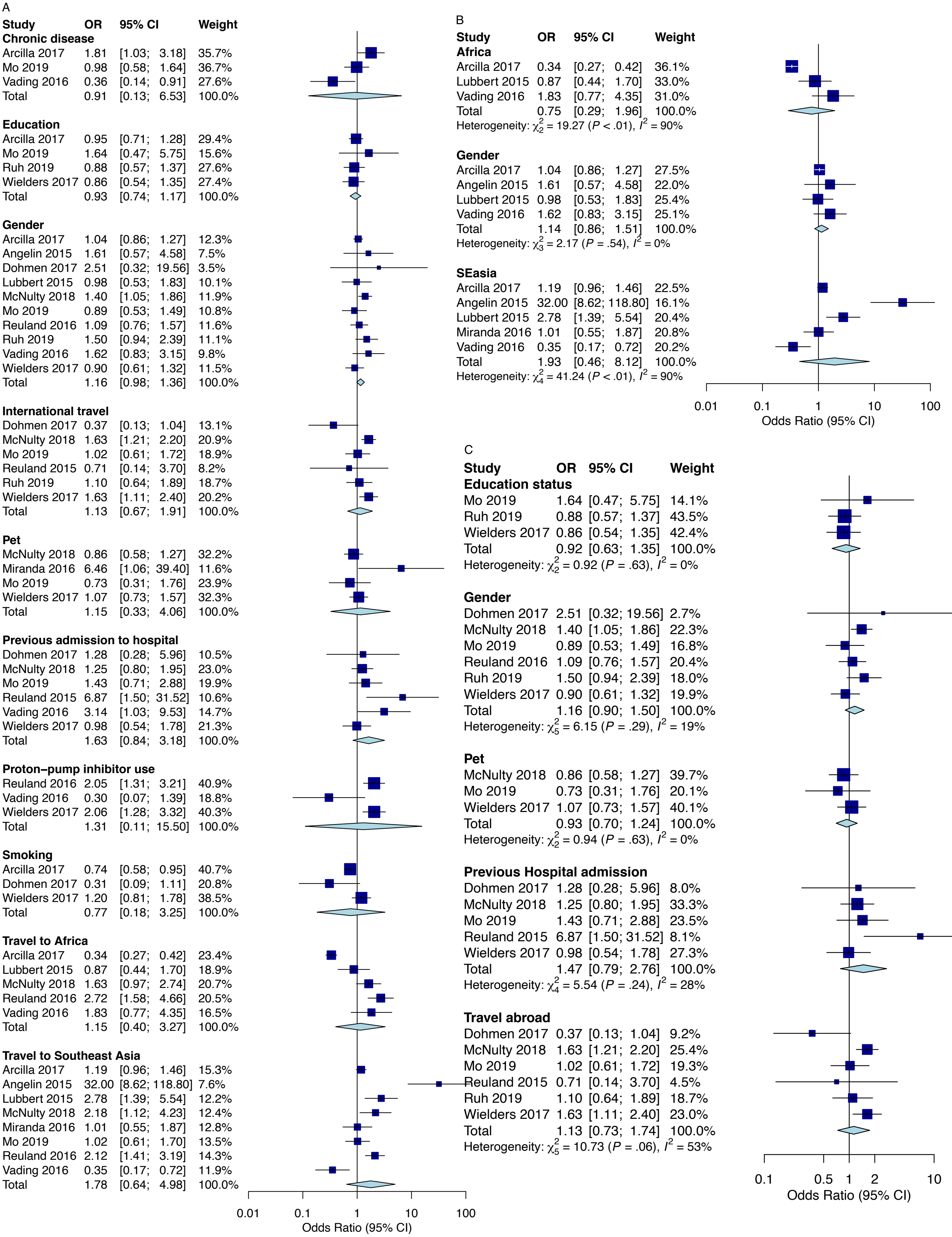
A
Study OR 95% CI Weight
Chronic disease
Arcilla 2017 1.81 [1.03; 3.18] 35.7%
Mo 2019 0.98 [0.58; 1.64] 36.7%
Vading 2016 0.36 [0.14; 0.91] 27.6%
Total 0.91 [0.13; 6.53] 100.0%
Education
Arcilla 2017 0.95 [0.71; 1.28] 29.4%
Mo 2019 1.64 [0.47; 5.75] 15.6%
Ruh 2019 0.88 [0.57; 1.37] 27.6%
Wielders 2017 0.86 [0.54; 1.35] 27.4%
Total 0.93 [0.74; 1.17] 100.0%
Gender
Arcilla 2017 1.04 [0.86; 1.27] 12.3%
Angelin 2015 1.61 [0.57; 4.58] 7.5%
Dohmen 2017 2.51 [0.32; 19.56] 3.5%
Lubbert 2015 0.98 [0.53; 1.83] 10.1%
McNulty 2018 1.40 [1.05; 1.86] 11.9%
Mo 2019 0.89 [0.53; 1.49] 10.8%
Reuland 2016 1.09 [0.76; 1.57] 11.6%
Ruh 2019 1.50 [0.94; 2.39] 11.1%
Vading 2016 1.62 [0.83; 3.15] 9.8%
Wielders 2017 0.90 [0.61; 1.32] 11.5%
Total 1.16 [0.98; 1.36] 100.0%
International travel
Dohmen 2017 0.37 [0.13; 1.04] 13.1%
McNulty 2018 1.63 [1.21; 2.20] 20.9%
Mo 2019 1.02 [0.61; 1.72] 18.9%
Reuland 2015 0.71 [0.14; 3.70] 8.2%
Ruh 2019 1.10 [0.64; 1.89] 18.7%
Wielders 2017 1.63 [1.11; 2.40] 20.2%
Total 1.13 [0.67; 1.91] 100.0%
Pet
McNulty 2018 0.86 [0.58; 1.27] 32.2%
Miranda 2016 6.46 [1.06; 39.40] 11.6%
Mo 2019 0.73 [0.31; 1.76] 23.9%
Wielders 2017 1.07 [0.73; 1.57] 32.3%
Total 1.15 [0.33; 4.06] 100.0%
Previous admission to hospital
Dohmen 2017 1.28 [0.28; 5.96] 10.5%
McNulty 2018 1.25 [0.80; 1.95] 23.0%
Mo 2019 1.43 [0.71; 2.88] 19.9%
Reuland 2015 6.87 [1.50; 31.52] 10.6%
Vading 2016 3.14 [1.03; 9.53] 14.7%
Wielders 2017 0.98 [0.54; 1.78] 21.3%
Total 1.63 [0.84; 3.18] 100.0%
Proton–pump inhibitor use
Reuland 2016 2.05 [1.31; 3.21] 40.9%
Vading 2016 0.30 [0.07; 1.39] 18.8%
Wielders 2017 2.06 [1.28; 3.32] 40.3%
Total 1.31 [0.11; 15.50] 100.0%
Smoking
Arcilla 2017 0.74 [0.58; 0.95] 40.7%
Dohmen 2017 0.31 [0.09; 1.11] 20.8%
Wielders 2017 1.20 [0.81; 1.78] 38.5%
Total 0.77 [0.18; 3.25] 100.0%
Travel to Africa
Arcilla 2017 0.34 [0.27; 0.42] 23.4%
Lubbert 2015 0.87 [0.44; 1.70] 18.9%
McNulty 2018 1.63 [0.97; 2.74] 20.7%
Reuland 2016 2.72 [1.58; 4.66] 20.5%
Vading 2016 1.83 [0.77; 4.35] 16.5%
Total 1.15 [0.40; 3.27] 100.0%
Travel to Southeast Asia
Arcilla 2017 1.19 [0.96; 1.46] 15.3%
Angelin 2015 32.00 [8.62; 118.80] 7.6%
Lubbert 2015 2.78 [1.39; 5.54] 12.2%
McNulty 2018 2.18 [1.12; 4.23] 12.4%
Miranda 2016 1.01 [0.55; 1.87] 12.8%
Mo 2019 1.02 [0.61; 1.70] 13.5%
Reuland 2016 2.12 [1.41; 3.19] 14.3%
Vading 2016 0.35 [0.17; 0.72] 11.9%
Total 1.78 [0.64; 4.98] 100.0%
0.01 0.1 1 10 100
Odds Ratio (95% CI)
B
Study OR 95% CI Weight
Africa
Arcilla 2017 0.34 [0.27; 0.42] 36.1%
Lubbert 2015 0.87 [0.44; 1.70] 33.0%
Vading 2016 1.83 [0.77; 4.35] 31.0%
Total 0.75 [0.29; 1.96] 100.0%
Heterogeneity: χ^2_2 = 19.27 (P < .01), I^2 = 90%
Gender
Arcilla 2017 1.04 [0.86; 1.27] 27.5%
Angelin 2015 1.61 [0.57; 4.58] 22.0%
Lubbert 2015 0.98 [0.53; 1.83] 25.4%
Vading 2016 1.62 [0.83; 3.15] 25.1%
Total 1.14 [0.86; 1.51] 100.0%
Heterogeneity: χ^2_3 = 2.17 (P = .54), I^2 = 0%
SEasia
Arcilla 2017 1.19 [0.96; 1.46] 22.5%
Angelin 2015 32.00 [8.62; 118.80] 16.1%
Lubbert 2015 2.78 [1.39; 5.54] 20.4%
Miranda 2016 1.01 [0.55; 1.87] 20.8%
Vading 2016 0.35 [0.17; 0.72] 20.2%
Total 1.93 [0.46; 8.12] 100.0%
Heterogeneity: χ^2_4 = 41.24 (P < .01), I^2 = 90%
0.01 0.1 1 10 100
Odds Ratio (95% CI)
C
Study OR 95% CI Weight
Education status
Mo 2019 1.64 [0.47; 5.75] 14.1%
Ruh 2019 0.88 [0.57; 1.37] 43.5%
Wielders 2017 0.86 [0.54; 1.35] 42.4%
Total 0.92 [0.63; 1.35] 100.0%
Heterogeneity: χ^2_2 = 0.92 (P = .63), I^2 = 0%
Gender
Dohmen 2017 2.51 [0.32; 19.56] 2.7%
McNulty 2018 1.40 [1.05; 1.86] 22.3%
Mo 2019 0.89 [0.53; 1.49] 16.8%
Reuland 2016 1.09 [0.76; 1.57] 20.4%
Ruh 2019 1.50 [0.94; 2.39] 18.0%
Wielders 2017 0.90 [0.61; 1.32] 19.9%
Total 1.16 [0.90; 1.50] 100.0%
Heterogeneity: χ^2_5 = 6.15 (P = .29), I^2 = 19%
Pet
McNulty 2018 0.86 [0.58; 1.27] 39.7%
Mo 2019 0.73 [0.31; 1.76] 20.1%
Wielders 2017 1.07 [0.73; 1.57] 40.1%
Total 0.93 [0.70; 1.24] 100.0%
Heterogeneity: χ^2_2 = 0.94 (P = .63), I^2 = 0%
Previous Hospital admission
Dohmen 2017 1.28 [0.28; 5.96] 8.0%
McNulty 2018 1.25 [0.80; 1.95] 33.3%
Mo 2019 1.43 [0.71; 2.88] 23.5%
Reuland 2015 6.87 [1.50; 31.52] 8.1%
Wielders 2017 0.98 [0.54; 1.78] 27.3%
Total 1.47 [0.79; 2.76] 100.0%
Heterogeneity: χ^2_4 = 5.54 (P = .24), I^2 = 28%
Travel abroad
Dohmen 2017 0.37 [0.13; 1.04] 9.2%
McNulty 2018 1.63 [1.21; 2.20] 25.4%
Mo 2019 1.02 [0.61; 1.72] 19.3%
Reuland 2015 0.71 [0.14; 3.70] 4.5%
Ruh 2019 1.10 [0.64; 1.89] 18.7%
Wielders 2017 1.63 [1.11; 2.40] 23.0%
Total 1.13 [0.73; 1.74] 100.0%
Heterogeneity: χ^2_5 = 10.73 (P = .06), I^2 = 53%
0.1 0.5 1 2 10
Odds Ratio (95% CI)
